# Structure of melanins from the fungi *Ochroconis lascauxensis* and *Ochroconis anomala* contaminating rock art in the Lascaux Cave

**DOI:** 10.1038/s41598-017-13862-7

**Published:** 2017-10-18

**Authors:** José Maria De la Rosa, Pedro M. Martin-Sanchez, Santiago Sanchez-Cortes, Bernardo Hermosin, Heike Knicker, Cesareo Saiz-Jimenez

**Affiliations:** 1 Instituto de Recursos Naturales y Agrobiologia de Sevilla. IRNAS-CSIC. Avenida Reina Mercedes 10, 41012 Sevilla, Spain; 2 Instituto de Estructura de la Materia. IEM-CSIC. Serrano 121, 28006 Madrid, Spain

## Abstract

Two novel species of the fungal genus *Ochroconis*, *O*. *lascauxensis* and *O*. *anomala* have been isolated from the walls of the Lascaux Cave, France. The interest in these fungi and their melanins lies in the formation of black stains on the walls and rock art which threatens the integrity of the paintings. Here we report solid-state cross polarization magic-angle spinning ^13^C and ^15^N nuclear magnetic resonance (NMR) spectroscopy and surface-enhanced Raman spectroscopy (SERS) of the melanins extracted from the mycelia of *O*. *lascauxensis* and *O*. *anomala* in order to known their chemical structure. The melanins from these two species were compared with those from other fungi. The melanins from the *Ochroconis* species have similar SERS and ^13^C and ^15^N NMR spectra. Their chemical structures as suggested by the data are not related to 3,4-dihydroxyphenylalanine, 5,6-dihydroxyindole or 1,8-dihydroxynaphthalene precursors and likely the building blocks from the melanins have to be based on other phenols that react with the N-terminal amino acid of proteins. The analytical pyrolysis of the acid hydrolysed melanin from *O*. *lascauxensis* supports this assumption.

## Introduction

The microbiology of subterranean environments has received increasing attention due to the biodeterioration processes affecting the integrity of rock art and mural paintings^1,2^. Bacteria have colonised the rock art paintings in Altamira Cave, Spain^3^, and the mural paintings in Etruscan^4,5^ and Roman tombs^6,7^. Fungi have originated several outbreaks in Lascaux Cave, France^8,9^, and colonised Japanese tombs^10^. In this context, the identification of microorganisms involved in the biodeterioration of World Heritage Sites and the knowledge of the chemistry of their secondary metabolic products and melanins is of utmost interest for adopting conservation strategies.

One of the most evident sign of biodeterioration is the growth of dematiaceous fungi due to the black color of their melanins which appears on the colonised walls of monuments^1,10^. However, structural investigations of fungal melanins remain a challenging task due to their macromolecular and heterogeneous structure.

Melanins are located in the fungal cell wall which is composed of polysaccharides including β-linked glucan, chitin, mannan and galactofuran. Chitin is cross-linked to other cell wall polysaccharides and proteins and may form up to 40% of the fungal cell wall^11^. The protein content of some fungal chitin isolates ranged from 10 to15%^12^. Also up to 3% of lipids can be found in the cell walls^13^.

The detailed chemical structure of the fungal melanins is not known and microscopic studies showed that melanin granules are localised in the cell wall where they are likely cross-linked to polysaccharides components especially those containing mannose^11^. Therefore, it becomes evident that the extracted melanins contain cell wall polysaccharides, chitin, proteins and lipids, which makes it difficult to analyse “pure” melanin.

Classical works on melanins classified these macromolecular compounds as derived from 3,4-dihydroxyphenylalanine (DOPA) or 1,8-dihydroxynaphthalene (DHN) precursors^14^. In general, melanins are produced by the oxidation of DOPA, 5,6-dihydroxyindole, catechol and DHN. Polymerization of the precursors leads to melanin formation^14,15^.

However, some data pointed to the existence of other types of microbial extracellular melanins formed in culture media or even intracellularly by polymerisation of phenols and quinones in the presence of phenoloxidases^16^. Wheeler^17^ investigated the biosynthesis of melanins in different fungal species using the melanin inhibitor tricyclazole (5-methyl-1,2,4-triazolo-(3,4b)-benzothiazole). All but one of the 20 dark brown and black ascomycetous and anamorphic fungi apparently produced melanin from DHN. Nevertheless, melanin biosynthesis of *Aspergillus niger* was not affected by tricyclazole and it was suggested that this fungus probably uses alternative pathways.

Recently, two novel species of the melanised fungal genus *Ochroconis*, *Ochroconis lascauxensis* and *Ochroconis anomala* were isolated and described from black stains in Lascaux Cave, France. Despite their similar colony macro-morphologies and growth rates, both species showed to be clearly different between them and the closest related species *Ochroconis tshawytschae* and *Ochroconis anellii*, as revealed the molecular (ITS and RPB2 sequences) and micro-morphological (conidiophores and conidia) features^9^.

The black stains on the wall and ceiling limestone rocks were mainly produced by the accumulation of fungal melanins from *O*. *lascauxensis*, while the distribution of *O*. *anomala* in the cave was scarce. Surface-enhanced Raman spectroscopy (SERS) revealed the relationship between the black stains and the melanin of *O*. *lascauxensis* ^18^.

However, it appears that there could be other reasons for the formation of different and extensive black stains coating the surface of the clayey sediments, near the cave ground. In these sediments a high concentration of black Mn oxides was found and the data supported a biologically induced mineralization for the oxides.

Fungi and bacteria isolated from the cave were tested for Mn oxides precipitation and only *Acremonium nepalense*, a very abundant and metabolically active fungus in the clayey black stains was associated with the precipitation of abundant masses of birnessite. In fact, *A*. *nepalense* mycelia, originally white-coloured, turned dark brown due to the formation of aggregates of insoluble Mn oxides deposited on the mycelia. The formation of Mn oxides was absent in 17 cave fungi tested, which included *Ochroconis* and black yeast species and 13 bacteria, including six *Pseudomonas* spp. and a *Bacillus* sp.^19^.

Increasing our knowledge on the chemical structure of melanins will improve the possibility of adopting strategies to control the cave fungi and to avoid their melanisation processes. One tool, to reach this goal and to discern the chemical structures of these insoluble heterogenic mixtures is high-resolution solid-state NMR spectroscopy^20^. This and related NMR techniques have recently been used for the study of melanoma melanins^21^ but their application for the investigation of fungal melanins are scarce.

The combination of ^13^C biosynthetic labeling and solid-state NMR permitted the identification of key functional groups in the melanin of *Cryptococcus neoformans*, a human pathogenic fungus^22^. Furthermore Zhong *et al*.^23^ stated that the *C*. *neoformans* melanin contained components derived from sources other than L-DOPA polymerisation, suggesting that covalent linkages between L-DOPA-derived products and polysaccharide components may serve to attach the melanin to cell wall structures.

Prados-Rosales *et al*.^24^ found that the melanin from the edible mushroom *Auricularia auricula* showed NMR structural differences, relative to the *C*. *neoformans* melanin, with regard to the variable proportions of alkyl chains or oxygenated carbons. Different abundances of alkyl chains and oxygen functional groups were found in the^13^C NMR spectra of a number of melanins from soil fungi^20,25–27^. The melanins of these fungi (e.g. *Eurotium echinulatum*, *Epiccocum nigrum*, *Hendersonula toruloidea*, etc.) were compared with soil humic substances, as a part of these macromolecules was considered of fungal origin. It was shown that these fungi synthesised phenols that upon enzymatic oxidation reacted with proteins and/or peptides to form black polymers or melanins. These melanins were not based on DOPA or DHN precursors but their aromatic building blocks were related to phenols from the acetate-malonate pathway^16,28^. This was also supported by a negative test with tricyclazole or kojic acid for the fungus *E*. *nigrum* ^29^.

Another technique used for getting information on the chemical structure of macromolecules is SERS, a sensitive technique that results in the enhancement of Raman scattering by molecules adsorbed on rough metal surfaces. SERS is particularly useful for studies with a very limited quantity of sample. However, only a few melanins have been studied by SERS, namely the melanins from sepia^30^ and *O*. *lascauxensis* ^18^.

In the present work, the melanins from *O*. *lascauxensis* and *O*. *anomala* were subjected to solid-state ^13^C and ^15^N NMR and SERS spectroscopies. Due to the similarities in some spectroscopic characteristics we have compared the melanins from these two *Ochroconis* species with those from *Ochroconis tshawytschae*, a closely related species, and *Stachybotrys chartarum*, a fungus associated with water damage indoors and production of mycotoxins. In addition, the acid hydrolysed melanin of *O*. *lascauxensis* was pyrolysed in order to provide insight into the precursors. Analytical pyrolysis has become an important tool for the characterization of complex carbonaceous matrices because most of the pyrolysis products have a well-known origin, thus they yield valuable fingerprint information on the molecular structure of macromolecular substances^6^.

## Results and Discussion

The fungus *Ochroconis lascauxensis* contributes to the formation of black stains on the walls of the Lascaux Cave^1,8,9,18^. The melanin of this fungal species as well as those from other related species: *O*. *anomala* and *O*. *tshawytschae* have not been investigated and their chemical composition is unknown.

### Elemental analysis

The elemental composition of the melanins from *O*. *lascauxensis* and *O*. *tshawytschae* and their atomic ratios are shown in Table 1. The C, H and N content of the *Ochroconis* melanins are in the range of those obtained for the melanins of *Aspergillus niger*, *Stachybotrys chartarum* ^31^ and *Coprinus* spp.^32^. However, the S content of *Ochroconis* melanins is two to three times lower than those of *A*. *niger* and *S*. *chartarum*. The O content is similar to those of *A*. *niger* and *Coprinus* spp. but higher than that of *S*. *chartarum*. The *O*. *anomala* melanin has C and N contents in the range of other fungal melanins.

**Table 1:** Elemental analyses of *Ochroconis* spp. and *Stachybotrys chartarum* melanins.

Melanins	%C	%H	%N	%S	%O	H/C*	O/C*
*Ochroconis lascauxensis*	55.2 ± 1.4	6.2 ± 0.2	5.7 ± 0.3	0.5 ± 0.1	32.4 ± 2.0	1.35	0.44
*Ochroconis tshawytschae*	54.6 ± 1.2	6.2 ± 0.1	6.9 ± 0.5	0.5 ± 0.1	31.8 ± 1.9	1.36	0.59
*Ochroconis anomala*	56.5 ± 1.5	n.d.	5.6 ± 0.4	n.d.	n.d.		
Stachybotrys chartarum**	57.3	7.0	6.8	1.2	27.7	1.47	0.36

*Atomic ratios. **Data from Schnitzer *et al*.^31^. n.d. no determined. (S.D. for n = 3).

Atomic H/C and O/C ratios can be used to elucidate structural formulae of melanins. H/C measures the degree of aromaticity of melanins^32^. The H/C ratios of the *Ochroconis* melanins are consistent with a model polymer having as structural unit a benzene nucleus and an aliphatic chain of three C atoms (e.g. model C_3_-alkylbenzene, H/C: 1.33), which indicate a relative aliphatic character. O/C ratio is an indicator of the carbohydrate and carboxylic acid contents. The O/C values in the range of 0.44–0.59 point to a relatively important O-alkyl and carboxylic acid composition.

*Stachybotrys chartarum* melanin has a higher aliphaticity (e.g. model C_5_-alkylbenzene, H/C: 1.45) and a lower O/C than the *Ochroconis* melanins. The fungus *S*. *chartarum* synthesises in addition to phenols^16^, a high number of mycotoxins, among which are the sesquiterpenoid trichothecenes (e.g. H/C: 1.33, O/C: 0.40) and the diterpenoid atranones (e.g. H/C: 1.33, O/C: 0.33). These terpenoids contain different carbonyl and hydroxyl groups^33^, which likely would form the macromolecule.

According to Prados-Rosales *et al*.^24^ fungal melanins include 5,6-dihydroxyindole and 5,6-dihydroxyindole-2-carboxylic acid monomeric units with 6–9% N or 1,8-dihydroxynaphthalene with no N in its structure. If we assumed this statement, the melanins from *Ochroconis* and *Stachybotrys* should be considered indole-derived melanins. This is not coincident with the ^15^N NMR spectra, which show that the N in the fungal melanins derives from proteins/peptides attached to the macromolecule and not from N heterocycles^20^.

### Surface-Enhanced Raman Spectroscopy

**Figure 1:**
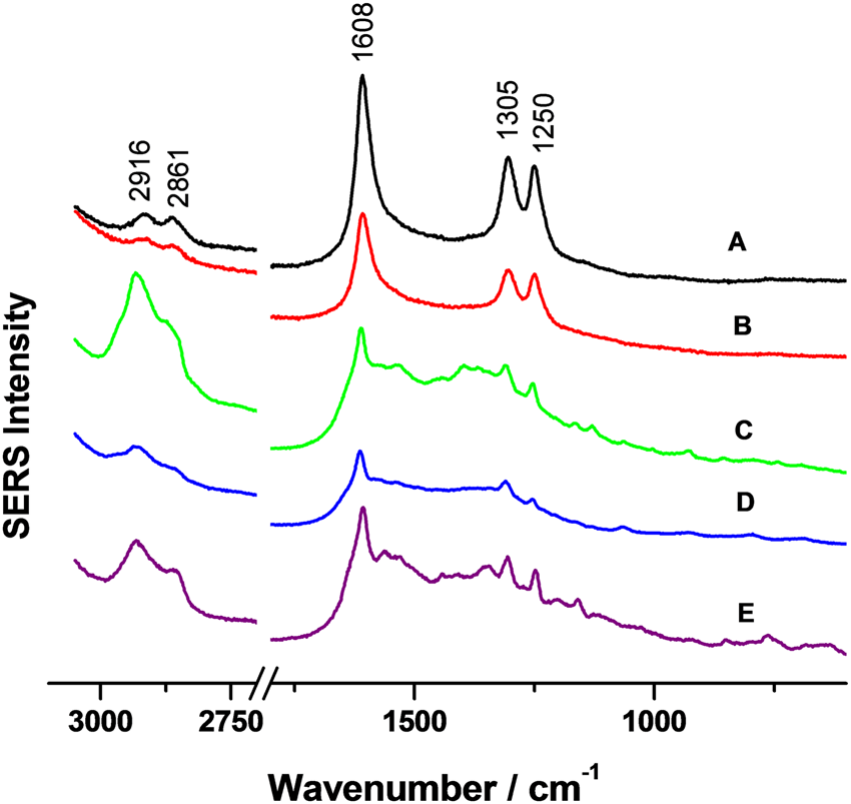
SERS spectra. (**A**) *Ochroconis lascauxensis* melanin. (**B**) *Ochroconis tshawytschae* melanin. (**C,D** and **E**) Black stains from Lascaux Cave, France. (**C**) From the Bull Hall, (**D**) From the Axial Gallery. (**E**) From the Nave.

The spectra of the *O*. *lascauxensis* and *O*. *tshawytschae* melanins are depicted in Fig. 1A and B. They show strong bands at 1608, 1305 and 1250 cm^−1^, which are attributed to C=O, C–C stretching vibrations in aromatic compounds and to C–O stretching vibrations of hydroxyl groups, respectively^18^. These three bands can be considered as markers of *Ochroconis* melanins and are also found in the black stains collected from Lascaux Cave (Fig. 1C–E). Bands with minor intensities were also observed at 2916 and 2861 cm^−1^. This is the region of C–H stretching of aliphatic chains^34^. Compared to the bands of aromatic components, those of aliphatic chains are less intense because of the high Raman resonance effect of the aromatic moieties^18^.

**Figure 2:**
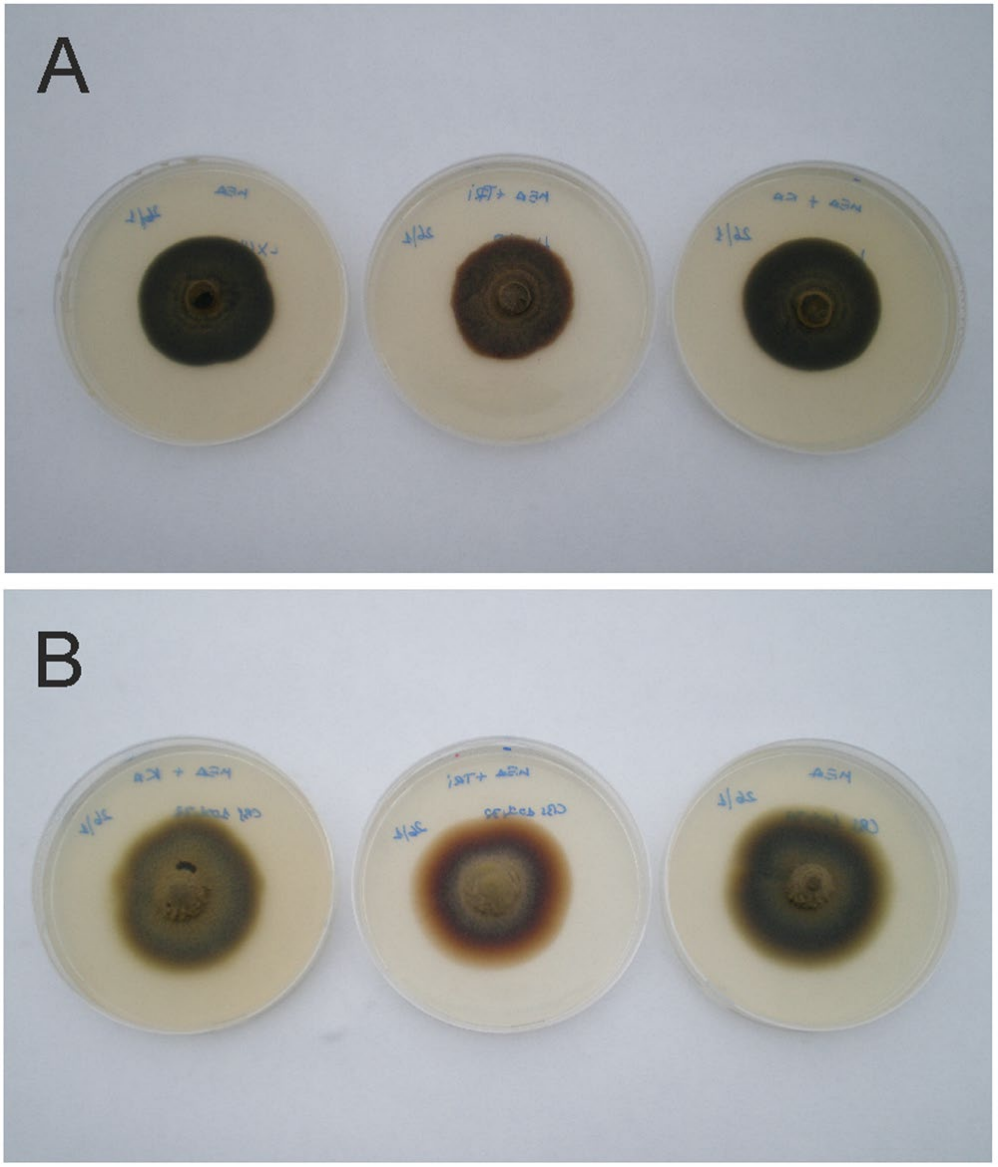
Inhibition test of melanin synthesis. Plates after 36 days culture. (**A**) *Ochroconis lascauxensis*. (**B**) *Ochroconis tshawytschae*. Plate position: control of malt extract agar (MEA) without inhibitor (left), MEA and DHN melanin inhibitor (tricyclazole) (center) and MEA and DOPA melanin inhibitor (kojic acid) (right).

The SERS spectra of *Ochroconis* melanins differ from that obtained for sepia melanin, a DOPA melanin^30^. In fact, in the spectra of fungal melanins no bands assigned to pyrrole and indole ring vibrations are observed. This can be explained by taking into account that in contrast to *Ochroconis* melanins, sepia melanin is formed by the polymerisation of 5,6-dihydroxyindole. As confirmed by the SERS spectra, *Ochroconis* melanins have not DOPA nor 5,6-dihydroxyindole precursors. This was also sustained by the behaviour of *Ochroconis* spp. in the presence of melanin inhibitors^35^. Kojic acid did not inhibit the production of *Ochroconis* melanin which supports that its synthesis is unlikely to occur by DOPA polymerisation reactions. Furthermore, the fact that tricyclazole was not able to inhibit melanin synthesis denotes that the *Ochroconis* melanin is not based on DHN precursors^29^ (Fig. 2).

The SERS spectrum of *S*. *chartarum* melanin was published by Martin-Sanchez *et al*.^18^ and resulted to be different, with a number of bands that cannot be found in *Ochroconis*. The unique coincident band was at 1609 cm^−1^ (attributed to C=O stretching vibrations in aromatic structures).

The SERS spectra of the black stains collected from the cave walls contained the three characteristic bands present in the spectra of *Ochroconis* melanins, which add evidence to the participation of *O*. *lascauxensis* in the black stain formation. In addition, other small bands were observed, which can be assigned to the presence of polysaccharides, proteins, lipids, etc. from microbial cells (bacteria and other fungi) inhabiting the black stains^1^. An assignment of bands to these macromolecules in bacterial surfaces was published by Neugebauer *et al*.^36^ and most of them were coincident with those of black stains.

### ^13^C NMR spectroscopy

**Figure 3:**
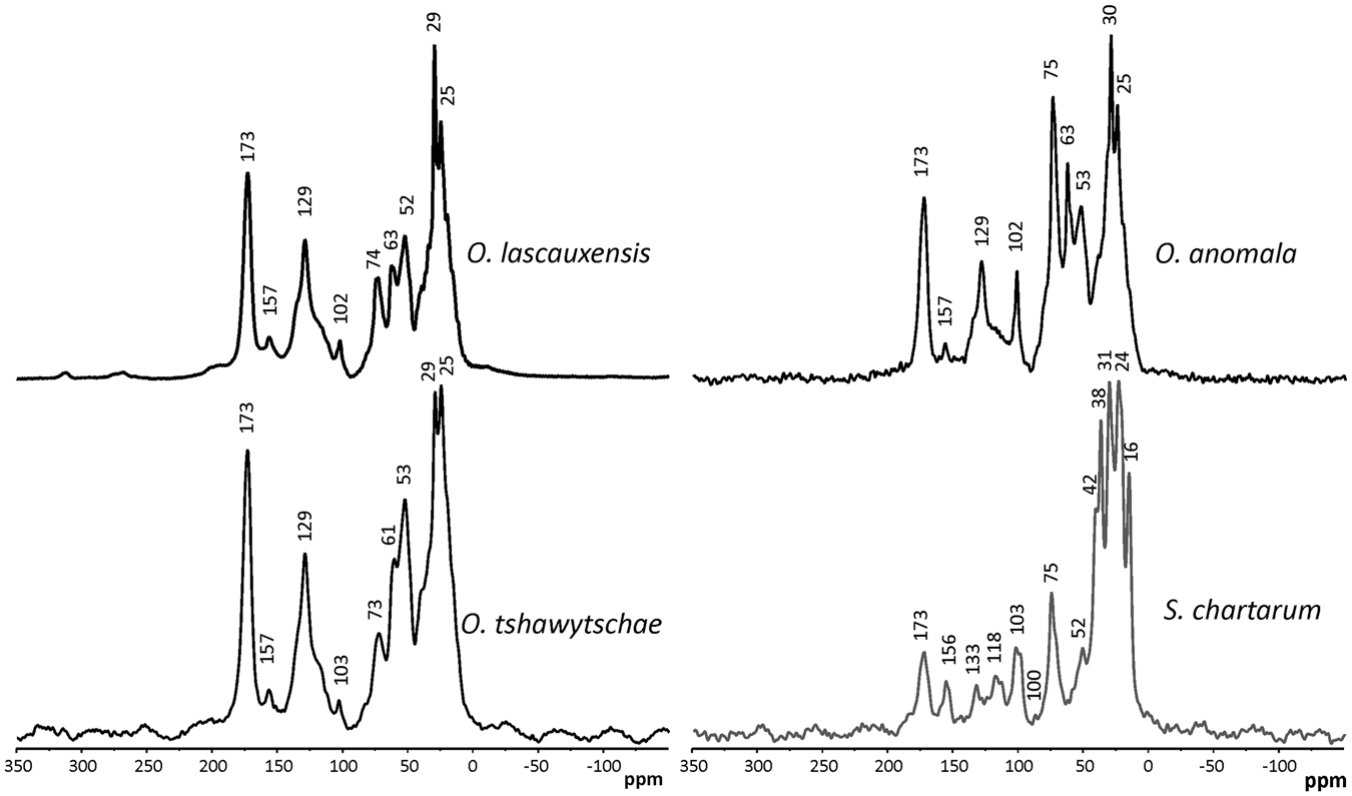
Solid state ^13^C NMR spectra of *Ochroconis lascauxensis, Ochroconis anomala, Ochroconis tshawytsc* *ha* *e*, and *Stachybotrys chartaru* *m* melanins.

The solid-state cross polarization magic-angle spinning (CP-MAS) ^13^C NMR spectra of the *Ochroconis* melanins are displayed in Fig. 3 and the corresponding integration of the signals for each C region is shown in Table 2.

**Table 2:** Relative intensity in solid state ^13^C NMR spectra of *Ochroconis* spp. and *Stachybotrys chartarum* melanins for each chemical shift region.

Melanins	Chemical shift (ppm)						
	245–185	185–160	160–140	140–110	110–60	60–45	45–0
*Ochroconis lascauxensis*	1.8	14.2	4.2	15.8	14.6	11.6	37.8
*Ochroconis anomala*	1.0	10.2	2.7	11.1	28.1	12.6	34.4
*Ochroconis tshawytschae*	1.2	14.6	3.1	14.6	12.9	14.7	39.0
*Stachybotrys chartarum*	2.5	6.8	3.6	8.1	16.9	7.1	55.2

The three *Ochroconis* melanins resulted in ^13^C NMR spectra with signals at comparable chemical shifts. The spectrum of *O*. *lascauxensis* shows intense signals in the chemical shift region between 0 and 45 ppm (peaks at 25 and 29 ppm), typically assigned to alkyl C. The peak at 24–25 ppm is assigned to terminal methyl (aliphatic side groups). Methyl C in *β-*position in amino acids and in medium and long alkyl chains has its resonance line at around 30 ppm. Signals of branched alkyl C in *α*-position are expected between 30 and 40 ppm. All spectra of the *Ochroconis* melanins show comparable relative abundance of alkyl C, from 34.4 to 39.0% of the total ^13^C intensity (Table 2). The peak at 52–53 ppm corresponds most likely to N-alkyl C^37^. Two peaks (at around 61–63 and 73–75 ppm) are present within the O-alkyl C region (60–90 ppm) and are commonly assigned to carbohydrates. The origin of the peak at 75 ppm, which is especially intense in the *O*. *anomala* melanin, is best explained by C-2, C-3 and C-5 in polysaccharide structures. The signal at 63 ppm is attributed to C-6 carbons in polysaccharides. However, due to the relative low intensity of the signal around 74 ppm to the total ^13^C intensity of the spectra of *O*. *lascauxensis* and *O*. *tshawytschae*, this signal may have additional contributions, possibly from N-alkyl C in valine or proline-type structures.

The weak signal around 102–103 ppm, present in all *Ochroconis* melanins, could correspond to anomeric C-1 carbon^38^ and may also indicate non-protonated aromatic carbons^39^. The prominent peak at 129 ppm corresponds to unsubstituted aromatic carbon^40^. Substitution by C-groups shifts the signal towards higher chemical shifts. It is noteworthy that the aromatic resonances (110–140 ppm) range from 8 to 16% in the fungal melanins (Table 2). The sharp peak centered at 173 ppm in the carboxyl region is usually assigned to carboxyl C, but is also indicative of amide linkages in peptides and proteins^41^. The absence of signal at ca. 200 ppm may indicate the lack of ketonic C-double bond.

The *O*. *anomala* melanin revealed the highest contribution of carbohydrates, (O-alkyl C) with 28.1% of the total ^13^C intensity (Table 2), although alcohols, also contribute to this region (60–90 ppm). In this particular case, it is remarkable the strong signal that appeared at around 74 ppm. The melanin from *A*. *niger* also showed strong signals of carbohydrates due to the contribution of the polysaccharide nigeran^26^. The aromatic C region (110–140 ppm) with resonances at 129 ppm and the aromatic CO/CN region (140–160 ppm) with a resonance line peaking at 157 ppm, corresponds to 11.1% and 2.7% of the total ^13^C intensity, respectively (Table 2), which falls within the range expected for fungal melanins^25,26^. The low intensity between 140–160 ppm in the spectra of all melanins reflects a very low content of phenolic groups (≤4.2%). A similar result was previously reported by Lüdemann *et al*.^27^. Nevertheless, numerous authors neglected this finding and explained that the complex chemical structure may cause a shift in the position of the phenolic carbon signal.

The *O*. *tshawytschae* melanin exhibited a spectrum similar to that of *O*. *lascauxensis*. This is dominated by two intense peaks at 25 and 29 ppm (alkyl C) and resonances at 52–53 ppm (N-alkyl groups), 61–63 ppm (O-alkyl), 102–103 ppm (anomeric C-1 carbon), 129 ppm (aromatic C) and 173 ppm (carboxyl C).

If we compare the spectra of the melanins from *O*. *lascauxensis* and *O*. *anomala* it is evident that they have similar chemical components, although varying in total ^13^C intensity, namely the high carbohydrate content in *O*. *anomala*. On the contrary, the intensities of phenols and carboxyls are higher in *O*. *lascauxensis*. The *O*. *tshawytschae* spectrum is similar to that of *O*. *lascauxensis* but their N-alkyl and O-alkyl peaks are somewhat higher.

Particularly intriguing is the chemical structure of the melanin of *S*. *chartarum*. The spectrum of this melanin (Fig. 3) is analogous to that of *S*. *chartarum* melanin previously published by Gonzalez-Vila *et al*.^25^ and both spectra are similar to that reported by Lüdemann *et al*.^27^ for *Stachybotrys atra* melanin. The fungal species *S*. *atra* and *S*. *chartarum* are synonyms and consequently these authors refer to the same fungus. Therefore, the^13^NMR spectra of the melanin from different strains of *S*. *chartarum* suggest that they have analogous chemical structure, which is made up of aliphatic chains with a minor contribution of aromatic units^27^.

The ^13^C NMR spectrum of *S*. *chartarum* melanin shows notable differences in comparison with the spectra of *Ochroconis* melanins, as previously observed for the SERS spectrum. The spectrum of *S*. *chartarum* displays the highest intensity in the alkyl-C region (55.2%) and a lower intensity in the aromatic region (8.1%). Peptide structures may be present. Nevertheless, the relative low signal intensity in the chemical shift region assigned to carboxyl C suggests that medium chain alkyl C units represent an important constituent of this melanin^42^. The sharp and resolved peaks at 16, 24, 31, 38 and 42 ppm in the *S*. *chartarum* melanin indicate paraffinic carbons in specific and repeating configurations, as suggested by Hatcher *et al*.^43^ for marine humic acids. The presence of phenols may be supported by the signals at 156 ppm (O-aryl C), 118 ppm (C next to the O-aryl C) and 133 ppm (C-aryl C).

*S*. *chartarum* synthesises a variety of mycotoxins^33,44,45^. The ^13^C NMR data of this melanin agree with the possible involvement of mycotoxins (trichothecenes or atranones, among others), which possess a terpenoid structure having methylene chains and carbonyl groups. It can be speculated that the reactive groups from mycotoxins and/or phenols^16^ can undergo polymerisation reactions resulting in a complex macromolecule with a marked aliphatic character. Analytical pyrolysis of this melanin produced secondary pyrolysis products that resulted in the formation of series of alkylcyclohexenes, alkylbenzenes and alkylnaphthalenes from aliphatic structures^46^. Similar secondary reactions have been described in the pyrolysis of fatty acids which yield alkylbenzenes^47^.

### ^15^N NMR spectroscopy

**Figure 4:**
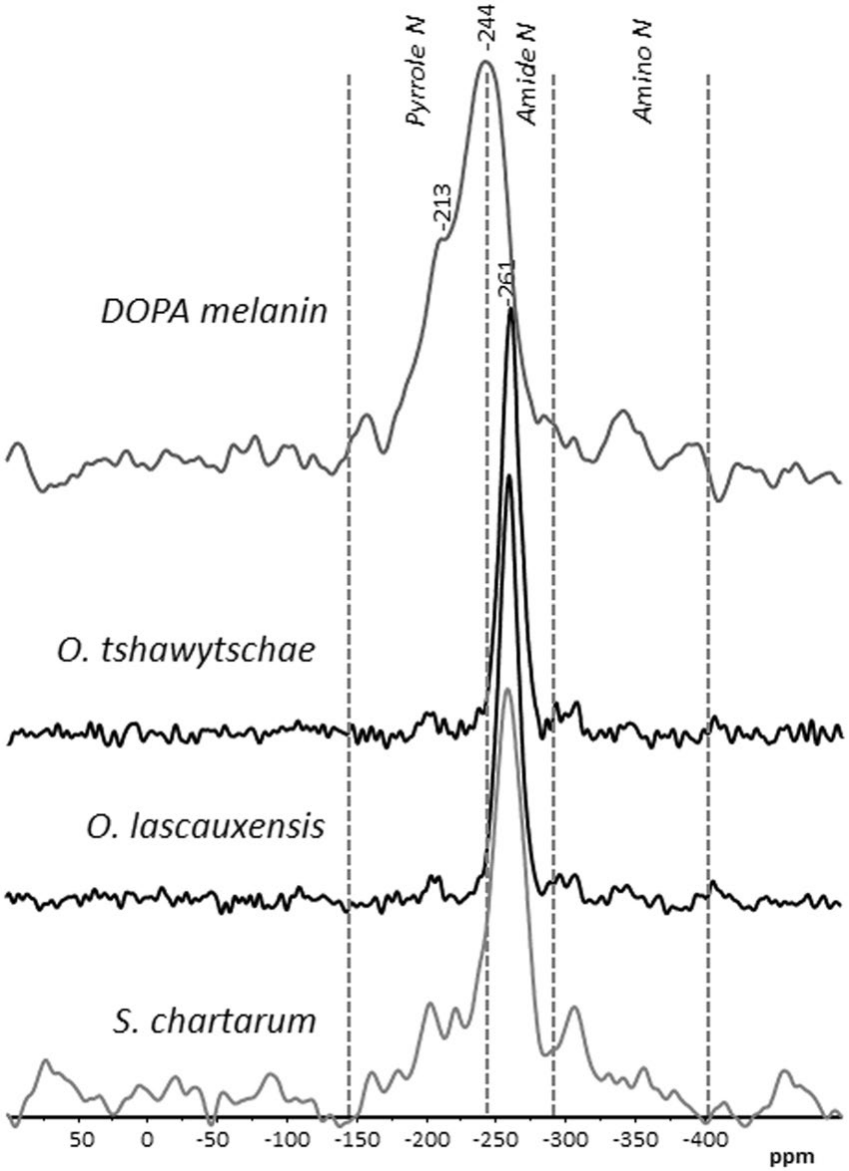
Solid state ^15^N NMR spectra of DOPA melanin and *Ochroconis tshawytschae*, *Ochroconis lascauxensis* and *Stachybotrys chartarum* melanins.

The elemental analyses of the fungal melanins have an N content from 5.6 to 6.9 (Table 1). With the assumptions that the N is attributable to proteins, and that in such structures the N content is 16%, its reciprocal 6.25 may be used for a rough estimation of protein content of melanins. Accordingly, the contribution of proteins ranges between 35 and 43.1% of the dry weight. To confirm the presence of proteins or peptides, solid-state CP-MAS ^15^N NMR spectroscopy was used. The respective spectra of the *Ochroconis* melanins show an intense signal at –261 ppm, in the peptide/amide region (−245 to −285 ppm; Fig. 4), which is best assigned to amide N^[20]^. The small signal at −345 ppm corresponds to terminal and free amino groups in peptides but in amino sugars. Further signals around −294 ppm and −306 ppm are detected and are best assigned to free –NH_2_ as it occurs in urea or amino acids such as glutamine or arginine. Some intensity is also observed around −200 ppm. It may derive from pyrrole-type structures including histidine. The pattern of the solid-state ^15^N NMR spectra of the fungal melanins is comparable to that observed for plants, algae and microbes^42,48^ which supports the conclusion that a major part of their organic N is bound in peptide structures.

In contrast, the solid-state ^15^N NMR spectrum of the DOPA melanin (Fig. 4) shows high intensity in the region from −145 to −245 ppm, which correspond to N in aromatic heterocycles of the indole- and/or pyrrole type^20,48^. Most melanin and humic acid models claim that indole- and/or pyrrole-derivatives are the major N-forms^14,49^. Nevertheless, in our study, heterocyclic N dominates the N forms in the DOPA melanin, but may play a minor role in the *S*. *chartarum* melanin. Comparable results were obtained previously^20^. Our results indicate that the presence of aromatic N heterocyclic compounds in *Ochroconis* melanins is neglectable, if any. This confirms that DOPA or 5,6-dihydroxyindole is not a constituent of the *Ochroconis* melanin, which agrees with the SERS data.

### Analytical pyrolysis of the acid hydrolysed melanin of *Ochroconis lascauxensis*

Acid hydrolysis is a method used to purify fungal melanins^24^. It has been reported that this procedure removes polysaccharides, proteins, lipids and loosely held compounds from soil humic acids^50,51^. Analytical pyrolysis have been applied to geo- and biopolymers to investigated their complex macromolecular structures^46,52–54^.

In order to shed more light on the backbone, the melanin of *O*. *lascauxensis* was pyrolysed at 500 °C. The melanin was previously acid hydrolysed with 6 *N* HCl, washed and dialysed in water, dried and the residue subjected to a preheating at 300 °C in a micro-furnace to remove lipids and other residual or loosely held compounds.

**Figure 5:**
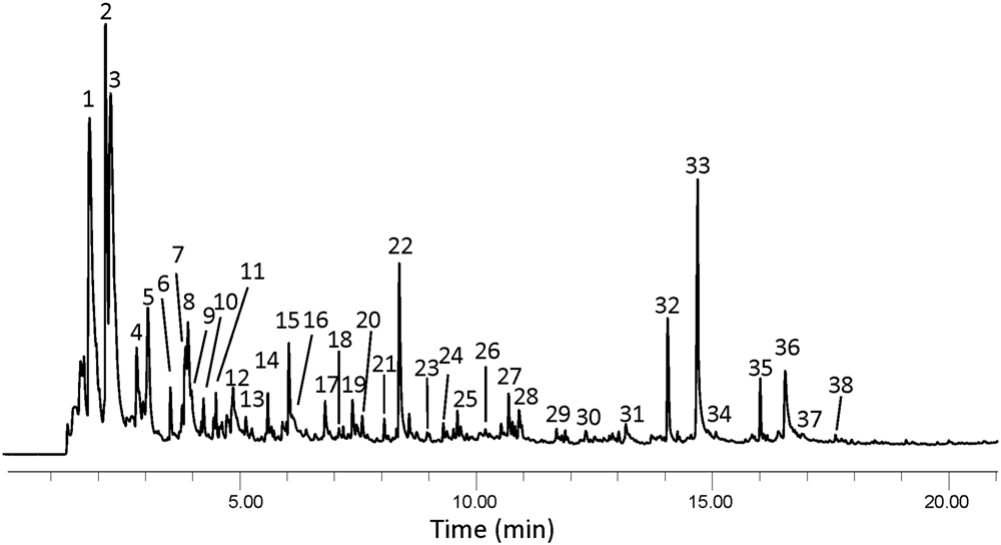
Chromatogram from the acid hydrolysed melanin of *Ochroconis lascauxensis* pyrolysed at 500 °C. Peaks refer to Table 3.

Figure 5 shows the chromatogram and Table 3 the major and/or representative pyrolysis products. The pyrolysate of this sample was characterised by the intensity of a few compounds such as benzene (1), pyridine (2), toluene (3), *n*-ethylbenzene (4), styrene (5), phenol (7), benzonitrile (8), naphthalene (15), biphenyl (22), hexadecanenitrile (32) and hexadecanoic acid (33). Other relevant compounds were the series of *n*-alkylbenzenes from C_3_ to C_6_ (peaks 6, 11, 14 and 17), methylphenol (12), methylnaphthalene (19), *n*-hexadecene (27), octadecanenitrile (35) and octadecanoic acid (36).

**Table 3:** Major and/or representative pyrolysis products from the acid hydrolysed *Ochroconis lascauxensis* melanin pyrolysed at 500 °C.

Peak	Compounds	Peak	Compounds
1	Benzene	20	Methylnaphthalene
2	Pyridine	21	*n-*Heptylbenzene
3	Toluene	22	Biphenyl
4	*n-*Ethylbenzene	23	Dimethylnaphthalene
5	Styrene	24	*n-*Octylbenzene
6	*n-*Propylbenzene	25	*n-*Pentadecane
7	Phenol	26	Phenylphenol
8	Benzonitrile	27	*n-*Hexadecene
9	Benzofuran	28	Fluorene
10	Indane	29	*n-*Decylbenzene
11	*n-*Butylbenzene	30	Methylfluorene
12	Methylphenol	31	Phenanthrene
13	Methylbenzofuran	32	Hexadecanenitrile
14	*n-*Pentylbenzene	33	Hexadecanoic acid
15	Naphthalene	34	Phenylnaphthalene
16	Benzoic acid	35	Octadecanenitrile
17	*n-*Hexylbenzene	36	Octadecanoic acid
18	*n-*Hexylthiophene	37	*n*-Docosene
19	Methylnaphthalene	38	*n-*Tricosene

Minor pyrolysis products were a few members of the series of *n*-alkylbenzenes from C_7_ to C_10_ (peaks 21, 24 and 29), alkylnaphthalenes (peaks 20 and 23), fluorene and methylfluorene (peaks 28 and 30), phenanthrene (31), benzofuran and methylbenzofuran (peaks 9 and 13), *n*-hexylthiophene (18), representative of the series of *n*-alkylthiophenes that elutes after the corresponding *n*-alkylbenzene, benzoic acid (16), phenylphenol (26), phenylnaphthalene (34), etc.

Most of the pyrolysis products obtained from the melanin have a definite origin. It is known that the alkylnitriles are formed via dehydratation of alkylamides^55^, which are typically produced by reaction between the ammonia evolved from the pyrolysis of proteins and the fatty acids that contain the extracted melanin. These pyrolysis compounds indicated that acid hydrolysis did not remove all melanin N and the same can be applied to the fatty acids. In this way, the most abundant fatty acids (hexadecanoic and octadecanoic acids) yield the corresponding alkylnitriles. The high peak of pyridine and the presence of benzonitrile are common in the pyrolysis of proteins and peptides^54^.

The homologous series of *n*-alkylbenzenes and *n*-alkylthiophenes were artifacts generated during pyrolysis from unsaturated fatty acids. It has been proved that under the same analytical conditions used to study humic acids (soil macromolecule with similar components that some fungal melanins, e.g. fatty acids and sulfur, among others), thermal cyclisation and aromatisation of fatty acids take place in presence of either elemental or organic sulfur, and the identification of *n*-alkylbenzenes (as major compounds), *n*-alkylthiophenes and *n*-alkylnaphthalenes in pyrolysates of soil humic substances could be related to thermal reactions of aliphatic precursors^56–58^.

*n*-Alkanes are commonly produced by fungi and a homologous series of *n*-alkanes up to C_33_ was identified^59^. Homologous series of *n*-alkanes and *n*-alkenes have been found in the pyrolysate of *Ochroconis* melanin. These two series of compounds were also identified in the pyrolysis of fatty acids^56^.

Polycyclic aromatic compounds (naphthalene, fluorene, phenanthrene) and its alkyl derivatives have been found in the pyrolysates of different soil humic fractions, microbial biomass, etc.^54,60,61^. A possible origin due to re-arrangement and cyclisation upon pyrolysis of the abundant aliphatic compounds present in the extracted melanins, due to the elevated temperature used, cannot be discarded.

It could be claimed a DHN structure for the fungal melanin by the presence of naphthalene and a few alkylnaphthalenes among the pyrolysis products. First at all, the identification and relatively low abundance of these compounds in the pyrolysate of an acid hydrolysed melanin (from which were removed the polysaccharides and most of the proteins, lipids, etc.) do not support the existence of a polymeric DHN backbone because a higher intensity of peaks related to the naphthalene core should be expected. In addition, naphthalene, biphenyl, fluorene and other polycyclic aromatic hydrocarbons are commonly found in the pyrolysates of soil humic substances^54,60^, algal biomass^61^, geo- and biomacromolecules without a definite DHN structure, and in the combustion of biomass^54^.

It is remarkable that besides phenol, many other phenolic compounds were identified in the melanin pyrolysate, although in minor amounts. Phenolic compounds were obtained in high amounts in the pyrolysis of *E*. *echinulatum* melanin^62^ that is formed extracellularly by oxidation of phenols^28^. Very strong signals in the aromatic region (25% of the total intensity) were evidenced in the ^13^C NMR spectrum of this melanin^20^ while about 16% was noticed in the *O*. *lascauxensis* melanin (Table 2). This suggests that phenols were also involved in the formation of the *Ochroconis* melanin core, as discussed previously for other fungi^16,28^. Briefly, the process has been described as follows: phenols are readily oxidised to quinones by phenoloxidases. Phenols and quinones interact with proteins reversibly by hydrogen bonding or irreversibly by covalent bonding. Phenolic compounds and the N-terminal amino acid are linked together by oxidative coupling. This results in the formation of extracellular black macromolecules or intracellular melanins^16,28^. The reaction products of phenols with amino acids are stable against acid hydrolysis. Using peptides it was found that all amino acids, except the N-terminal which is bound to oxidised phenols, could be hydrolysed^63^.

Melanoidins, high molecular weight heterogeneous black polymers formed by reaction of sugars and amino acids through the Maillard reaction were also considered as a possible mechanism of *Ochroconis* melanin formation. Pyrolysis of melanoidins yield pyrazines, pyridines, pyrroles, oxazoles and furans^64^. In addition,^13^C and ^15^N solid-state NMR spectra of melanoidins^65^ were very different from those shown here for the fungal melanins and no evidence of an extended N-containing aromatic network chemically bound to a polyfuran network, known to be the main components of the melanoidins^66^, could be found. In melanoidins based on fatty acids and proteins, pyrrole formation and polymerisation mechanism were also contributing to the nonenzymatic browning reaction^67^.

### *Ochroconis* vs *Stachybotrys* melanins

The melanins from the three different *Ochroconis* species have similar SERS and ^13^C and ^15^N NMR spectra. Their chemical structure as suggested by the data is not related to 3,4-dihydroxyphenylalanine, 5,6-dihydroxyindole or 1,8-dihydroxynaphthalene precursors, but likely on other phenolic building blocks.

In comparison with the ^13^C NMR spectra of *Ochroconis* melanins the *Stachybotrys* melanin shows lower aromaticity and carboxyl/carbonyl C contents (Fig. 3 and Table 2), which is consistent with the elemental analyses.

Carbohydrates moiety is high in the melanin of *O*. *anomala* and the lowest quantity is found in the melanin of *Stachybotrys*. As revealed by solid-state ^15^N NMR spectroscopy peptide structures comprise the dominant N-form in *S*. *chartarum* but pyrrole-type N forms cannot be neglected (Fig. 4).

Thus, the melanins extracted from *Ochroconis* mycelia are made up of mixtures of complex cell wall materials, with different contributions of polysaccharides, proteins, lipids and aromatic compounds as denoted the ^13^C NMR and ^15^N NMR spectra (Figs 3 and 4). Analytical pyrolysis of the acid hydrolysed melanin of *O*. *lascauxensis* produced a high number of secondary pyrolysis products (alkylbenzenes, alkylthiophenes, alkylnaphthalenes, alkylphenanthrenes, etc.) which origin cannot be allocated to a DHN core but to artifacts produced by the pyrolysis of fatty acids and other aliphatic compounds (Fig. 5 and Table 3). The identification of phenols in the pyrolysate could tentatively support the contribution of phenols and proteins to the formation of the melanin core, to which is attached other cell wall components.

## Conclusions

*Ochroconis lascauxensis* and *Ochroconis anomala* are two novel species of dematiaceous fungi isolated from the walls of the Lascaux Cave, France. Their melanins contribute to the formation of black stains on the walls and rock art. In order to discern the chemical structure of these two melanins, solid-state cross polarization magic-angle spinning ^13^C and ^15^N nuclear magnetic resonance (NMR) spectroscopy and surface-enhanced Raman spectroscopy (SERS) have been performed and compared with the melanin of a closely related species: *Ochroconis tshawytschae*, and that of *Stachybotrys chartarum*. All the extracted melanins have different contributions of cell wall materials, including polysaccharides, proteins, lipids and aromatic compounds.

The melanins from the three *Ochroconis* species showed similar spectra, denoting a common origin but different from that of *S*. *chartarum* melanin. Furthermore, the lack of signals corresponding to pyrrole or indole rings (SERS spectroscopy) added to the absence of aromatic N heterocyclic compounds in the NMR spectra of *Ochroconis* melanins indicated that they are not related to the precursors typically attributed to the chemical structure of fungal melanins: 3,4-dihydroxyphenylalanine (DOPA) or 5,6-dihydroxyindole. In addition, no clear evidence of the 1,8-dihydroxynaphthalene (DHN) precursor was found.

The pyrolysis products released from the acid hydrolysed melanin of *O*. *lascauxensis*, which would correspond to the melanin core, supported the previous assumption. The origin of the secondary pyrolysis products identified (alkylbenzenes, alkylthiophenes, alkylnaphthalenes, alkylphenanthrenes, etc.) cannot be allocated to a DHN backbone but to artifacts produced by the pyrolysis of cell wall components, resistant to acid hydrolysis. Also phenolic compounds linked to an N-terminal amino acid are resistant to acid hydrolysis, as denoted the presence of nitrogen derivatives among the pyrolysis products. Therefore, the chemical structures of *Ochroconis* melanins have to be based on phenols that react with the N-terminal amino acid of proteins.

There is no report on the genomes of the three *Ochroconis* spp. here studied. However, the *in silico* genome analysis of an *Ochroconis mirabilis* ^68^ strain revealed the presence of potential genes that enable the fungus to synthesise melanin via the 1,8-dihydroxynaphthalene (DHN) pathway and to produce trichothecenes and the sulfur-containing amino acid taurine^69^. If these genes are included in the genome of our *Ochroconis* spp. or if polyketide synthase (PKS) genes are involved in the synthesis of *Ochroconis* melanins merit further investigations. Therefore, the chemical structure of the *Ochroconis* melanins is still in discussion.

## Methods

### Fungi

The fungi *Ochroconis lascauxensis* CBS131815^T^ and *Ochroconis anomala* CBS131816^T^ were isolated from the Lascaux Cave walls^9^. *Ochroconis tshawytschae* CBS 100438^T^, a closely related fungus, was obtained from the reference fungal CBS-KNAW collection at the Westerdijk Fungal Biodiversity Institute, Utrecht, The Netherlands. The fungi were cultured in BD Bacto malt extract (20 g·L^−1^) liquid medium at 22 °C in an orbital shaker (150 rpm) for one month. The resulting biomass was filtered with double sterile gauze and subsequently homogenised in sterile distilled water using a Krups blender at maximum speed for 5 min. The protocols used for melanin extraction and purification are shown in Supplementary Figure S1.

For acid hydrolysis the melanin was refluxed with 6 *N* HCl for 24 hours. This procedure removes polysaccharides, proteins, lipids and loosely held compounds^50,51^. After acid hydrolysis the residual melanin was washed, dialysed in water and dried.

The melanin from *Stachybotrys chartarum* ^31^ was provided by Dr. Morris Schnitzer. A hamster melanoma melanin (DOPA-melanin)^70^ was also analysed for comparison purposes. Inhibition test of melanins (Fig. 2) were carried out on solid malt extract agar medium (20 g·L^−1^ BD Bacto malt extract, 20 g·L^−1^ agar) with 30 mg·L^−1^ of tricyclazole or kojic acid, and the corresponding controls without any melanin inhibitor. The plates were incubated in darkness at 22 °C for 36 days.

### Elemental analysis

Elemental analysis (C, H, O, N, S) affords a general characterization of the melanins. In this study, C, N, H and S contents were determined in triplicate using an elemental analyser (Carlo-Erba EA-1100-CHNS microanalyser). O content was estimated as the difference between the C, H, N and S concentrations.

### Raman measurements

Surface-enhanced Raman spectroscopy analyses were done by using hydroxylamine Ag nanoparticles prepared by reduction with hydroxylamine as described by Martin-Sanchez *et al*.^18^. SERS spectra were registered with a Renishaw Raman RM2000, equipped with a charge-coupled device (CCD) camera, using the line at 514 nm of an Ar^+^ laser as excitation source. Resulting spectra are the average of 10 scans at 5 s and using a laser power at the sample of 2 mW^18^.

### Nuclear Magnetic Resonance Spectroscopy

The solid-state ^13^C NMR spectra were obtained with a Bruker Avance III HD 400 MHz instrument operating at a frequency of 100.64 MHz and using a triple resonance probe for ZrO_2_ rotors of 4 mm OD with Kel-F caps. The cross polarization (CP) technique was applied during magic-angle spinning (MAS) of the rotor at 14 kHz and the spectra were acquired with a ramped 1H-pulse during a 1 ms contact time to circumvent Hartmann-Hahn mismatches. Employing a pulse delay of 300 ms, between 10,000 and 40,000 scans were accumulated. A line broadening of 50 Hz was used. The ^13^C chemical shifts were calibrated relative to tetramethylsilane (0 ppm) with glycine (COOH at 176.08 ppm). The spectra were quantified by subdividing them into the following chemical shift regions as described by Knicker^71^: alkyl C (0–45 ppm); N-alkyl/methoxyl C (45–60 ppm); O-alkyl C (60–110 ppm); aromatic C (110–160 ppm); carbonyl/amide C (160–245 ppm). The ^13^C intensity distribution was determined by integrating signal intensity over the above-mentioned chemical shift regions using the MestreNova 10 software.

The solid -state CPMAS ^15^N NMR spectra were obtained with the same instrument using a double resonance probe for ZrO_2_ rotors of 7 mm OD, which were spun at the magic angle at 6 kHz. A ramped 1H-pulse was applied during the contact time of 1 ms. Around 20,000 and 40,000 scans were accumulated using a pulse delays between 100 and 250 ms. After Fourier transformation, the spectra were edited with a line broadening of 50 Hz. The chemical shift was standardised to the nitromethane scale (0 ppm) and adjusted with ^15^N-labeled glycine (–347.6 ppm) according to Witanowski *et al*.^72^. Due to the very limited amount of sample and the size of the rotor used for ^15^N NMR spectroscopy, we were not able to analyse the melanin of *O*. *anomala*.

### Analytical pyrolysis

The melanin of *O*. *lascauxensis*, previously hydrolysed with 6 *N* HCl for 24 hours^50,51,73^, was deposited on small crucible capsules. Pyrolysis-gas chromatography/mass spectrometry was performed using a double-shot pyrolyser (Frontier Labs. model 2020i) attached to a GC/MS system Agilent 6890 N, as described elsewhere^6^. Briefly, a dry sample (~1 mg) was introduced into a preheated (300 °C) micro-furnace and then pyrolysed at 500 °C. The compounds evolved were then directly injected into the GC/MS for analysis. The gas chromatograph was equipped with a HP-5ms-UI capillary column. The detector consisted of an Agilent 5973 mass selective detector, and mass spectra were acquired at 70 eV ionizing energy. Compound assignments were achieved by single-ion monitoring (SIM) and by comparison with mass spectra libraries (NIST11 and Wiley7).

## Electronic supplementary material

Protocol for melanin extraction
