# Supplementary material for: Structure of melanins from the fungi Ochroconis lascauxensis and Ochroconis anomala contaminating rock art in the Lascaux Cave
Source: Sci Rep. 2017 Oct 18;7:13441. doi: 10.1038/s41598-017-13862-7 (PMC5647350; doi:10.1038/s41598-017-13862-7)
Supplement: Supplementary file 1 — Protocol for melanin extraction [file 41598_2017_13862_MOESM1_ESM.pdf]

Supplementary Information

Structure of melanins from the fungi *Ochroconis lascauxensis* and *Ochroconis anomala* contaminating rock art in the Lascaux Cave

Jose Maria de la Rosa[1], Pedro M. Martin-Sanchez[1], Santiago Sanchez-Cortes[2], Bernardo Hermosin[1], Heike Knicker[1], Cesareo Saiz-Jimenez[1]*

[1]Instituto de Recursos Naturales y Agrobiologia. IRNAS-CSIC. Avenida Reina Mercedes 10, 41012 Sevilla, Spain

[2]Instituto de Estructura de la Materia. IEM-CSIC. Serrano 121, 28006 Madrid, Spain

*Correspondence should be addressed to C.S.J. (email: saiz@irnase.csic.es)

Protocol for melanin extraction. The strains of *Ochroconis* were cultured in 250 ml Erlenmeyer flasks containing malt extract medium. Each flask was inoculated with 2 ml of a mycelial suspension. The flasks were incubated at 22°C under agitation (150 rpm) for one month (Fig. S1A). The resulting biomass (200-400 g fresh weight) was filtered and homogenised 5 minutes at full speed in distilled water using a Krups blender (Fig. S1B,C).

For melanin extraction, 5 *N* NaOH was added to the mixture until reaching a concentration of 0.5 *N* NaOH (Fig. S1D). The flasks were shaken for 24 hours, centrifuged at 5,000 rpm for 5 minutes, and the supernatant containing the dissolved melanin was recovered by filtration (Fig. S1E).

The residual biomass was re-suspended again in 0.5 *N* NaOH. This extraction process was repeated several times and all the supernatants obtained were acidified with 12 *N* HCl to pH 1.5 until precipitation of the melanin. The precipitate was recovered by centrifugation at 5,000 g and washed twice with distilled water (Fig. S1F,G).

Subsequently, to remove the chloride ions from the precipitate a dialysis was carried out with successive changes of distilled water for 5 days (Fig. S1H) until a negative reaction of Cl^- with a solution of $AgNO_3$ was obtained. Finally, the resulting melanin was dried at 30°C and stored at room temperature (Fig. S1I).

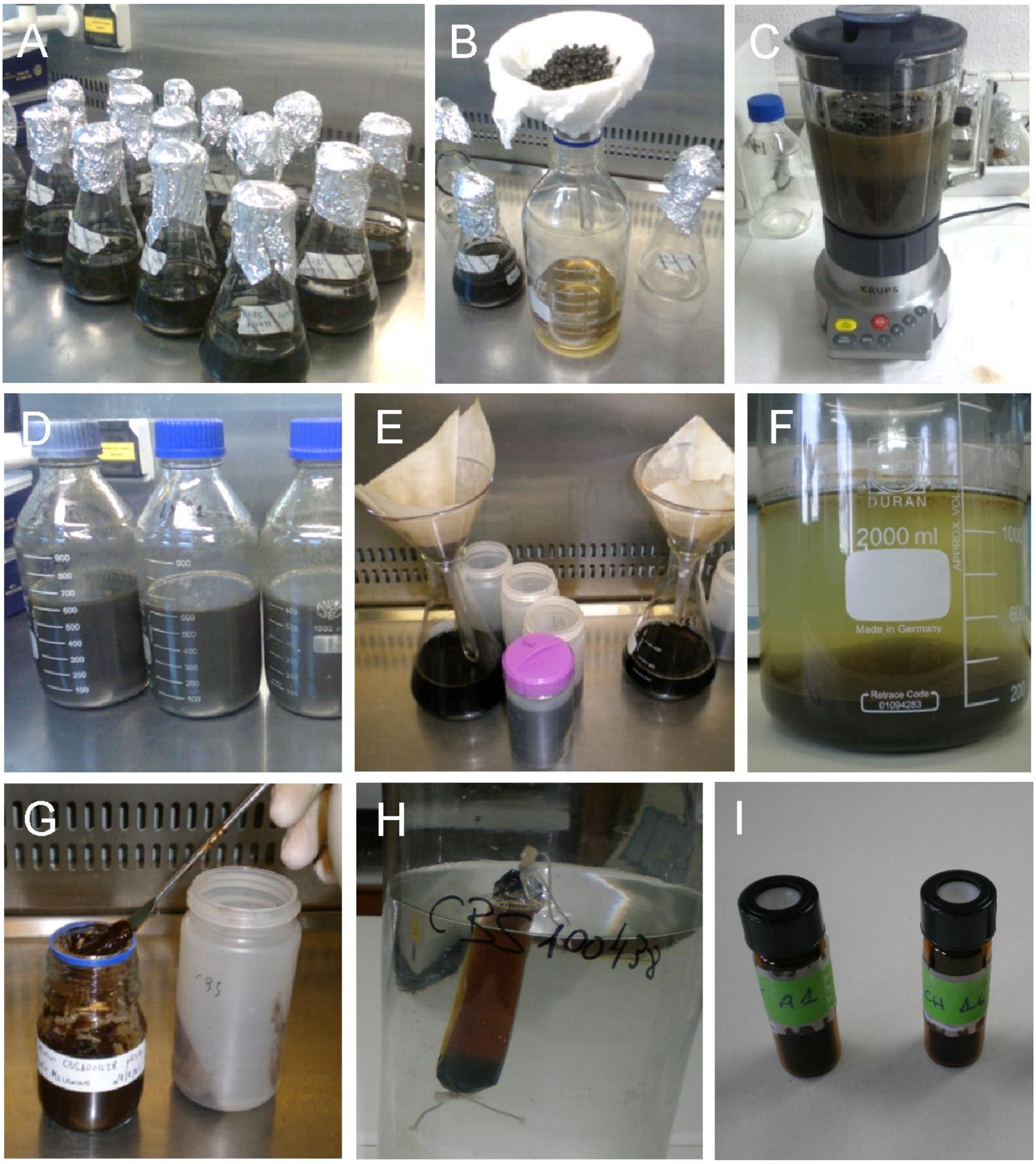

Figure S1. Melanin extraction from *Ochroconis* spp. (A) One month-old cultures in malt extract broth. (B) Recovering of biomass after incubation. (C) Biomass homogenisation in sterile distilled water. (D) Melanin extraction with 0.5 *N* NaOH. (E) Filtration of the melanin-containing supernatant. (F) Melanin precipitation by HCl at pH 1.5. (G) Melanin recovering after centrifugation. (H) Removal of chloride ions by dialysis. (I) Stored melanin after drying at 30°C.
